# Sleep disturbances in obsessive-compulsive disorder: influence of depression symptoms and trait anxiety

**DOI:** 10.1186/s12888-021-03038-z

**Published:** 2021-01-14

**Authors:** Cinto Segalàs, Javier Labad, Neus Salvat-Pujol, Eva Real, Pino Alonso, Sara Bertolín, Susana Jiménez-Murcia, Carles Soriano-Mas, Carmen Monasterio, José M. Menchón, Virginia Soria

**Affiliations:** 1 Department of Psychiatry, Bellvitge University Hospital. Bellvitge Biomedical Research Institute (IDIBELL), Neurosciences Group - Psychiatry and Mental Health, Feixa Llarga s/n. 08907, L’Hospitalet de Llobregat, Barcelona, Spain; 2 Centro de Investigación Biomédica en Red de Salud Mental (CIBERSAM), Carlos III Health Institute, Madrid, Spain; 3 Department of Clinical Sciences, School of Medicine, Universitat de Barcelona, Barcelona, Spain; 4 Department of Mental Health, Consorci Sanitari del Maresme. Institut d’Investigació i Innovació Parc Taulí(I3PT), Barcelona, Spain; 5 Centro de Investigación Biomédica en Red de Fisiopatología Obesidad y Nutrición (CIBEROBN), Carlos III Health Institute, Madrid, Spain; 6 Department of Psychobiology and Methodology of Health Sciences, Universitat Autònoma de Barcelona, Bellaterra, Spain; 7 Multidisciplinary Sleep Unit, Department of Respiratory Medicine, Bellvitge University Hospital. Bellvitge Biomedical Research Institute (IDIBELL), Section of Respiratory Medicine, L’Hospitalet de Llobregat, Barcelona, Spain; 8 Centro de Investigación Biomédica en Red de Enfermedades Respiratorias (CIBERES), Carlos III Health Institute, Madrid, Spain

**Keywords:** Obsessive-compulsive disorder, Sleep disorders, Sleep quality, Delayed sleep phase disorder, Depression, Trait anxiety

## Abstract

**Background:**

Sleep disturbances have been reported in obsessive-compulsive disorder (OCD) patients, with heterogeneous results. The aim of our study was to assess sleep function in OCD and to investigate the relationship between sleep and the severity of obsessive-compulsive (OC) symptoms, depressive symptoms and trait anxiety.

**Methods:**

Sleep quality was measured in 61 OCD patients and 100 healthy controls (HCs) using the Pittsburgh Sleep Quality Index (PSQI). Multiple linear regression was conducted to explore the association between sleep and psychopathological measures; a mediation analysis was also performed.

**Results:**

OCD patients showed poor sleep quality and more sleep disturbances compared to HCs. The severity of depression, trait anxiety and OC symptomatology were correlated with poor sleep quality. Multiple linear regression analyses controlling for potential confounders revealed that the severity of depression and trait anxiety were independently related to poor sleep quality in OCD. A mediation analysis showed that both the severity of trait anxiety and depression mediate the relationship between the severity of OC symptoms and poor sleep quality among patients with OCD.

**Conclusions:**

Our findings support the existence of sleep disturbances in OCD. Trait anxiety and depression play a key role in sleep quality among OCD patients.

**Supplementary Information:**

The online version contains supplementary material available at 10.1186/s12888-021-03038-z.

## Background

Sleep is crucial to brain function and important for maintaining cognitive and emotional processes [1]. Sleep disturbances have been reported in mood, eating, anxiety, personality, autism and schizophrenia disorders [2]. In major depressive disorder (MDD), insomnia or hypersomnia are considered one of the DSM-5 diagnostic criteria, and specifically, some sleep patterns measured by polysomnography (PSG), such as a decrease in slow-wave sleep (SWS) production and disturbed rapid eye movement (REM) sleep regulation, have been proposed as biological markers for depression [3].

Sleep disturbances are also prevalent in obsessive-compulsive disorder (OCD), as up to 48% of patients report these disturbances [4]. Previous research in OCD suggests that there is an association between specific sleep behaviours and clinical characteristics such as the severity of obsessive-compulsive (OC) symptoms, treatment resistance and age of onset of the disorder [5, 6]. Similarly, correlations between sleep disturbances, OC symptoms and response inhibition have been observed in subclinical samples [7, 8]. On the other hand, some studies argue that sleep disturbances in OCD could be attributed to the presence of comorbid depression [9–11].

Sleep disturbances in OCD have been studied with different measures, including self-report, observer report and PSG. Findings in this area could be classified into different groups: sleep quantity and quality, sleep disorders and sleep architecture.

The most reported disturbances in OCD compared to healthy controls (HCs) are the reduction in sleep duration and efficiency and increased nighttime awakenings [12–14]. Moreover, poorer sleep quality has been associated with the severity of OCD symptoms [15]. In contrast, another study that compared sleep function among four groups of subjects: OCD with and without comorbid depression, depressed patients and HCs [9] reported no differences in sleep function between OCD patients without comorbid depression and HCs, while OCD patients with depression and depressed patients exhibited very similar sleep patterns. These findings suggest the influence of comorbid depression on sleep quantity and quality. In line with these observations, two recent meta-analyses [10, 11] revealed that comorbid depression is a key factor in the existence of sleep disturbances in OCD, except for shortened sleep duration and longer sleep latency, which seem to be independent of comorbid depression.

Delayed sleep phase disorder (DSPD), which is considered a circadian sleep disorder (DSM-5), is a persistent inability to fall asleep coupled with extreme difficulty awakening in the morning. Several studies reported a higher prevalence of DSPD in OCD (17.6–42%) compared to the general population (0.2–10%) [5, 6, 16]. OCD patients with DSPD had more severe OC symptomatology and younger age at onset than patients without DSPD [5, 6]. The influence of depression on sleep circadian rhythm is unclear. Bobdey et al. [9] described DSPD in a subgroup of OCD patients without depression, supporting DSPD as a characteristic sleep behaviour in OCD; however, a recent meta-analysis reported a significantly higher prevalence of DSPD in OCD compared to HCs, but this effect became nonsignificant after controlling for comorbid depression [11].

In relation to sleep architecture in OCD, several studies identified alterations in REM and no-REM phase [12, 13, 17]. However, the results of a recent meta-analysis only revealed significant findings in a no-REM phase, describing a statistically significant reduction in the time spent in stage-2 sleep in OCD compared to HCs [10].

As it is described above, several studies have reported the influence of intensity of depression and OC symptomatology on the sleep function of OCD patients. However, up to date, no association between anxiety and sleep behaviour in these patients has been reported. Anxious traits have been related to sleep disruptions in the general population and in individuals with anxiety disorders such as panic disorder and posttraumatic stress disorder [18]. Alterations in sleep architecture are associated with both s*tate anxiety* (defined as a temporary reaction to adverse events) and *trait anxiety* (a more stable personality feature defined as a constant individual difference related to the tendency to respond with concerns, troubles and worries to various situations) [19] in patients with sleep disorders. In particular, trait anxiety was associated with poor sleep quality in a non-clinical sample [20].

The aim of our study was to assess the differences in sleep pattern and quality in patients with OCD compared to HCs and to determine the influence of the severity of depressive, trait anxiety and OC symptoms on sleep function in the patient group. Our hypothesis was that OCD patients would show worse sleep function (poor sleep quality and DSPD) than controls. We also aimed to determine whether the severity of psychopathology (depression and trait anxiety) could mediate the relationship between the intensity of OC symptoms and sleep disturbances.

## Methods

### Participants

The sample comprised 161 participants: 61 outpatients with OCD and 100 HCs. All patients were recruited from consecutive admissions to the Obsessive-Compulsive Disorders Unit of Bellvitge University Hospital (L’Hospitalet de Llobregat; Barcelona). HCs were recruited from the same geographic area through advertisements. The exclusion criteria were as follows: a history of substance abuse and/or dependence (except nicotine); other DSM-IV-TR Axis I and II comorbid psychiatric disorders except for MDD; neurological disease (except tics); having suffered a head injury with loss of consciousness; severe medical conditions; mental retardation; a history of bipolar disorder; a history of psychotic episodes; and having undergone electroconvulsive therapy and/or neurosurgery.

Written informed consent was obtained from each participant after a complete description of the study, which was approved by the Comitè Ètic d’Investigació Clínica (CEIC) de l’Hospital Universitari de Bellvitge. The authors assert that all procedures described herein comply with the Helsinki Declaration of 1975 (revised in 2013).

### Clinical assessment

Patients were diagnosed by an experienced psychiatrist using the Mini-International Neuropsychiatric Interview (MINI) [21] and met the DSM-IV-TR diagnosis criteria for OCD. Twenty-six OCD patients (42.6%) showed depression comorbidity.

The current or past history of psychiatric or neurological disorders, treatment with psychotropic medication, substance dependence or abuse, and head injury were ruled out in HCs by a semi-structured interview conducted by an experienced psychiatrist. All HCs also scored below 7 on the 28-item Spanish adaptation of the Goldberg General Health Questionnaire (GHQ-28) [22].

Patients received psychopharmacological treatment at the time of the sleep assessment following their clinical needs (98.4% of the sample). The World Health Organization Anatomical Therapeutic Chemical Classification System [23] was used to recode the defined daily dose (DDD) of antidepressant medication. DDD can be defined as the assumed average maintenance dose per day for drug use for its main indication in adults. Antipsychotic and benzodiazepine treatments were also registered, and doses were recorded in chlorpromazine equivalents for antipsychotic drugs [24] and in diazepam equivalents for benzodiazepines (equivalent doses obtained from the Ashton Manual, available at https://www.benzo.org.uk/manual/index.htm).

Sociodemographic variables such as sex, age and educational level were collected for all participants, and age of onset of OCD was collected in the patient group.

### Symptom measures

Depression was measured with the 17-item Hamilton Depression Rating Scale (HDRS) [25], and trait-related anxiety was measured with the Trait Subscale of the State-Trait Anxiety Inventory (STAI) [26], in all the sample. The internal consistency of the HDRS in our sample was good (Cronbach’s alpha = 0.81). Both STAI-state and STAI-trait subscales showed excellent internal consistency (Cronbach’s alpha was 0.96 and 0.95, respectively). In the patient group, OCD severity was measured using the clinical version of the Yale-Brown Obsessive-Compulsive Scale (Y-BOCS) [27]. The internal consistency of the Y-BOCS was excellent (Cronbach’s alpha = 0.91).

### Sleep function measures

Sleep function was assessed using the *Pittsburgh Sleep Quality Index* (PSQI) [28]. The PSQI is a retrospective self-report questionnaire that evaluates sleep function in the previous month and includes twenty-four self-related items, although only nineteen were used to calculate sleep quality that resulted in seven components (subjective sleep quality, sleep latency, sleep duration, habitual sleep efficiency, sleep disturbances, use of sleeping medication and daytime dysfunction). Each of them scored equally on a 0–3 score (the higher the score, the worse sleep quality). These components are as follows. A global PSQI score > 5 suggests poor sleep quality. In addition, specific items that would be useful to evaluate early insomnia and sleep phase shifting (SPS) were assessed: 1) the average amount of time a subject spends between going to bed and falling asleep; 2) usual bedtime; and 3) usual getting-up time. SPS was measured with the same procedure as described by Bobdey et al. [9]: the midpoint between bedtime and getting-up time for each subject was obtained (for example, if an individual went to bed at 23:00 h and got up at 06:00, their midsleep point would be 02:30). Mid-sleep is a chronotype indicator, with people from the morning type reporting earlier mid-sleep times when compared to people from the evening type, who report later mid-sleep times.

The internal consistency of the PSQI in our sample was good (Cronbach’s alpha = 0.79).

### Statistical analysis

Data processing was performed using SPSS 21.0 (SPSS, IBM, USA). Differences in demographic, clinical and sleep variables between OCD patients and HCs were assessed by comparing the means using Student’s *t* test for continuous variables, the X^2^ test for a linear trend for ordinal variables and the X^2^ test for categorical variables. As sleep measures were not distributed normally, we used the Spearman correlation analysis to explore the relationship between continuous clinical variables and sleep function. Consequently, the global PSQI score was log transformed (ln) with the following formula: lnPSQI = ln (PSQI+ 1). A multiple linear regression was performed to explore the relationship between OCD and sleep function (lnPSQI) after adjusting for clinical variables. The transformed global PSQI score was considered the dependent variable. Other covariates (sex, age, the severity of depression, trait anxiety, obsessive-compulsive symptomatology, benzodiazepine treatment doses and comorbidity with MDD) were included as independent variables.

The Process macro for SPSS [29] was used to conduct a mediation analysis. This macro allows the inclusion of multiple mediators and covariates. In this mediation analysis, we decided to include the Y-BOCS score as the main independent variable because the severity of OCD might affect sleep function and can also be associated with other psychopathology variables (depressive symptoms and trait anxiety) that can affect sleep function as well. The global PSQI score (ln) was used as the dependent variable. Two potential mediators were considered: severity of depression (HDRS) and trait anxiety. The significance of the indirect effects in this model was tested by bootstrapping. In brief, bootstrapping is a nonparametric method based on resampling with replacement, which is performed many times. It allows the generation of the indirect effect and a confidence interval (CI) of the bootstrapped distribution. If zero is not in the confidence interval, then the researcher can be confident that the indirect effect is different from zero and mediation exists.

## Results

### Demographic and clinical characteristics

Table 1 describes the demographic and clinical characteristics of the sample. As expected, OCD patients reported more psychopathology symptoms than HCs. OCD patients with depression reported more depressive symptoms but no significant differences in trait anxiety or OC symptoms when compared with OCD patients without depression. No significant differences in antidepressant or antipsychotic treatment were found in patients with or without MDD (Table 2), although the latter group received more benzodiazepines.

**Table 1:** Demographic and clinical characteristics of the sample

	OCD		HC				
	**N**	**%**	**N**	**%**	**Statistic**	**df**	***P*** **value**
**Sex**							
**Male**	35	57.4	51	51	X^2^ = 0.61	1	0.43
**Female**	26	42.6	49	49			
	**Mean**	**SD**	**Mean**	**SD**	**Statistic**	**df**	***P*** **value**
**Age (years)**	42.87	11.32	44.03	14.91	*t* = 0.52	159	0.60
**Education (years)**	12.77	3.81	13.50	4.02	*t* = 1.14	159	0.26
**HAM-D-17**	8.10	5.22	0.69	1.11	*t* = −13.7	159	< 0.001*
**Y-BOCS**	22.37	5.40	–				
**STAI-Trait**	34.93	11.43	13.18	8.31	*t* = −13.94	159	< 0.001*
**STAI-State**	26.26	14.96	10.44	6.94	*t* = −9.04	156	< 0.001*

*Abbreviations*: *OCD* Obsessive-Compulsive Disorder, *HC* Healthy Controls, *SD* Standard Deviation, *HAM-D* Hamilton Depression Rating Scale, *Y-BOCS* Yale-Brown Obsessive Compulsive Scale, *STAI* State-Trait Anxiety Inventory

*P < 0.05

**Table 2:** Clinical variables and psychopharmacological treatment in OCD patients, with or without MDD comorbidity

	OCD without MDD (***N*** = 35)	OCD with MDD (***N*** = 26)	Statistic	df	***P*** value
**Clinical Variables**					
HAM-D-17	6.67 (4.22)	10.04 (5.87)	*t = −2.62*	59	0.01^*^
Y-BOCS	22.55 (6.06)	22.13 (4.46)	*t = 0.29*	59	0.77
STAI-Trait	35.11 (9.82)	34.7 (13.50)	*t = 0.14*	59	0.89
STAI-State	26.40 (14.40)	26.04 (16.11)	*t = 0.09*	59	0.93
**Psychopharmacological Treatment Doses (DDD)**					
SSRI	3 (1.80)	3.12 (2.44)	*t =* −0.21	59	0.83
Dual-action antidepressants	0.09 (0.35)	0.38 (0.91)	*t =* −1.72	59	0.09
TCA	0.66 (0.91)	0.65(0.90)	*t =* 0.07	59	0.95
Other antidepressants	0.01 (0.08)	0.04 (0.20)	*t =* −0.65	59	0.52
Benzodiazepines (diazepam equivalents mg/day)	3.47 (6.75)	8.44 (9.35)	*t =* −2.41	59	0.02^*^
Antipsychotics (chlorpromazine equivalents mg/day)	64.86 (116.22)	52.31 (109.70)	*t =* 0.43	59	0.67

Data are mean (SD). *Abbreviations*: *OCD* Obsessive-Compulsive Disorder, *MDD* Major Depressive Disorder, *HAM-D* Hamilton Depression Rating Scale, *Y-BOCS* Yale-Brown Obsessive Compulsive Scale, *STAI* State-Trait Anxiety Inventory, *DDD* defined daily doses, *SSRI* selective serotonin reuptake inhibitors, *TCA* tricyclic antidepressants

*P < 0.05

### Sleep quality

OCD patients reported poorer sleep quality than HCs (Table 3 and Table 4), with significant differences in sleep latency, sleep disturbance, use of sleeping medication and daytime dysfunction. OCD patients reported more time between going to bed and falling asleep as well as later times for getting up than HCs (Table 4). Regarding SPS, OCD patients showed significantly later mid-sleep points compared to HCs. No statistically differences in sleep quality measures and SPS were observed between OCD patients with and without MDD comorbidity, an additional table file shows this in more detail (Supplementary Table 1).

**Table 3:** Pittsburgh Sleep Quality Index: scores and components’ descriptive analyses

PSQI component	Score 0		Score 1		Score 2		Score 3		Statistic ͣ	df	*P* value
	OCD	HC	OCD	HC	OCD	HC	OCD	HC			
	N (%)	N (%)	N (%)	N (%)	N (%)	N (%)	N (%)	N (%)			
**Subjective sleep quality**	14 (23%)	35 (35%)	24 (39.3%)	54 (54%)	16 (26.2%)	8 (8%)	7 (11.5%)	3 (3%)	X^2^ = 11.98	3	0.001*
**Sleep latency**	16 (26.2%)	36 (36%)	15 (24.6%)	42 (42%)	11 (18%)	19 (19%)	19 (31.1%)	3 (3%)	X^2^ = 15.38	3	< 0.001*
**Sleep duration**	36 (59%)	66 (66%)	15 (24.6%)	25 (25%)	6 (9.8%)	7 (7%)	4 (6.6%)	2 (2%)	X^2^ = 2.12	3	0.145
**Habitual sleep efficiency**	32 (52.5%)	61 (61%)	13 (21.3%)	25 (25%)	8 (13.1%)	7 (7%)	8 (13.1%)	7 (7%)	X^2^ = 2.85	3	0.091
**Sleep disturbance**	1 (1.6%)	6 (6%)	31 (50.8%)	78 (78%)	25 (41%)	16 (16%)	4 (6.6%)	0 (0%)	X^2^ = 20.60	3	< 0.001*
**Use of sleeping medication**	35 (57.4%)	86 (86%)	4 (6.6%)	8 (8%)	2 (3.3%)	2 (2%)	20 (32.8%)	4 (4%)	X^2^ = 24.23	3	< 0.001*
**Daytime dysfunction**	11 (18%)	51 (51%)	20 (32.8%)	41 (41%)	17 (27.9%)	7 (7%)	13 (21.3%)	1 (1%)	X^2^ = 38.25	3	< 0.001*

*Abbreviations*: *OCD* Obsessive-Compulsive Disorder, *HC* Healthy Controls, *PSQI* Pittsburgh Sleep Quality Index

ͣ X^2^ for a linear trend. *P < 0.05

**Table 4:** Group mean scores on sleep schedule and global for OCD and HC

	OCD	HC	Statistic	df	*P* value
	Mean (*SD)*	Mean (*SD)*			
**PSQI GS**	8.5 (4.8)	4.6 (3.0)	*t* = −6.18	159	< 0.001*
**Time falling sleep** (min)	35.7 (37.2)	17.7 (18.3)	*t* = −4.1	159	< 0.001*
**Sleep bedtime** (h:min)	24:16 (1:25)	24:02 (1:22)	*t* = −1.06	159	0.29
**Getting-up bedtime** (h:min)	8:41 (1:43)	7:58 (1:27)	*t* = −2.81	159	0.005*
**Mid-sleep-point** (h:min)	4:31 (1:23)	4:03 (1:20)	*t* = −2.09	159	0.04*

*Abbreviations*: *OCD* Obsessive-Compulsive Disorder, *HC* Healthy Controls, *SD* Standard Deviation, *PSQI GS* Pittsburgh Sleep Quality Index Global Score, *h* hour, *min* minutes

*P < 0.05

### Correlation analyses

In OCD patients, higher scores on the PSQI, which reflect worse sleep quality, were positively associated with the severity of depression, trait anxiety and OC symptomatology (Table 5). No associations were found between the mid-sleep point, age at assessment and age of onset of OCD and sleep function and any clinical variables.

**Table 5:** Spearman’s correlations between clinical variables and sleep function in OCD patients and HCs

	OCD (61)	HC (100)
	PSQI	PSQI
**Age**	0.02	0.15
**Age of onset**	−0.07	–
**HDRS**	0.60**	0.32**
**Y-BOCS**	0.30*	**–**
**STAI-Trait**	0.56**	0.43**
**Mid-sleep-point**	−0.07	−0.2*

Data represent r coefficient values

*Abbreviations: OCD* Obsessive-Compulsive Disorder, *HCs* Healthy Controls, *PSQI* Pittsburgh Sleep Quality Index, *HDRS* Hamilton Depression Rating Scale, *Y-BOCS* Yale-Brown Obsessive Compulsive Scale, *STAI* State-Trait Anxiety Inventory

*P < 0.05

** *P* < 0.01

In HCs, poor sleep quality was positively associated with the severity of depression symptoms and trait anxiety and earlier mid-sleep points (morning chronotype) (Table 5).

### Predictors and mediating factors influencing sleep quality

The results of the multiple regression model for the global PSQI score are shown in Table 6. After adjusting for clinical variables (sex, age, the severity of depression, trait anxiety, obsessive-compulsive symptomatology, benzodiazepine treatment doses and comorbidity with MDD), only higher levels of depression and trait anxiety in OCD patients were associated with worse sleep quality.

**Table 6:** Results of the multiple linear regression analyses in relation to sleep function (PSQI scores)

	β	***P***-value	***R***^***2***^
**Independent variables**			0.41
Y-BOCS	0.08	0.529	
HDRS	0.35	0.025*	
STAI-Trait	0.31	0.032*	
Age	−0.07	0.574	
Female Sex	−0.09	0.446	
MDD	0.04	0.735	
BTD	0.06	0.632	

lnPSQI was considered the dependent variable

*Abbreviations*: *β* Standardized regression coefficient, *PSQI* Pittsburgh Sleep Quality Index, *Y-BOCS* Yale-Brown Obsessive Compulsive Scale, *HDRS* Hamilton Depression Rating Scale, *STAI-T* State-Trait Anxiety Inventory, *MDD* Major Depressive Disorder, *BTD* Benzodiazepine Treatment Doses, *lnPSQI* Log-transformed Pittsburgh Sleep Quality Index

*P < 0.05

**Fig. 1:**
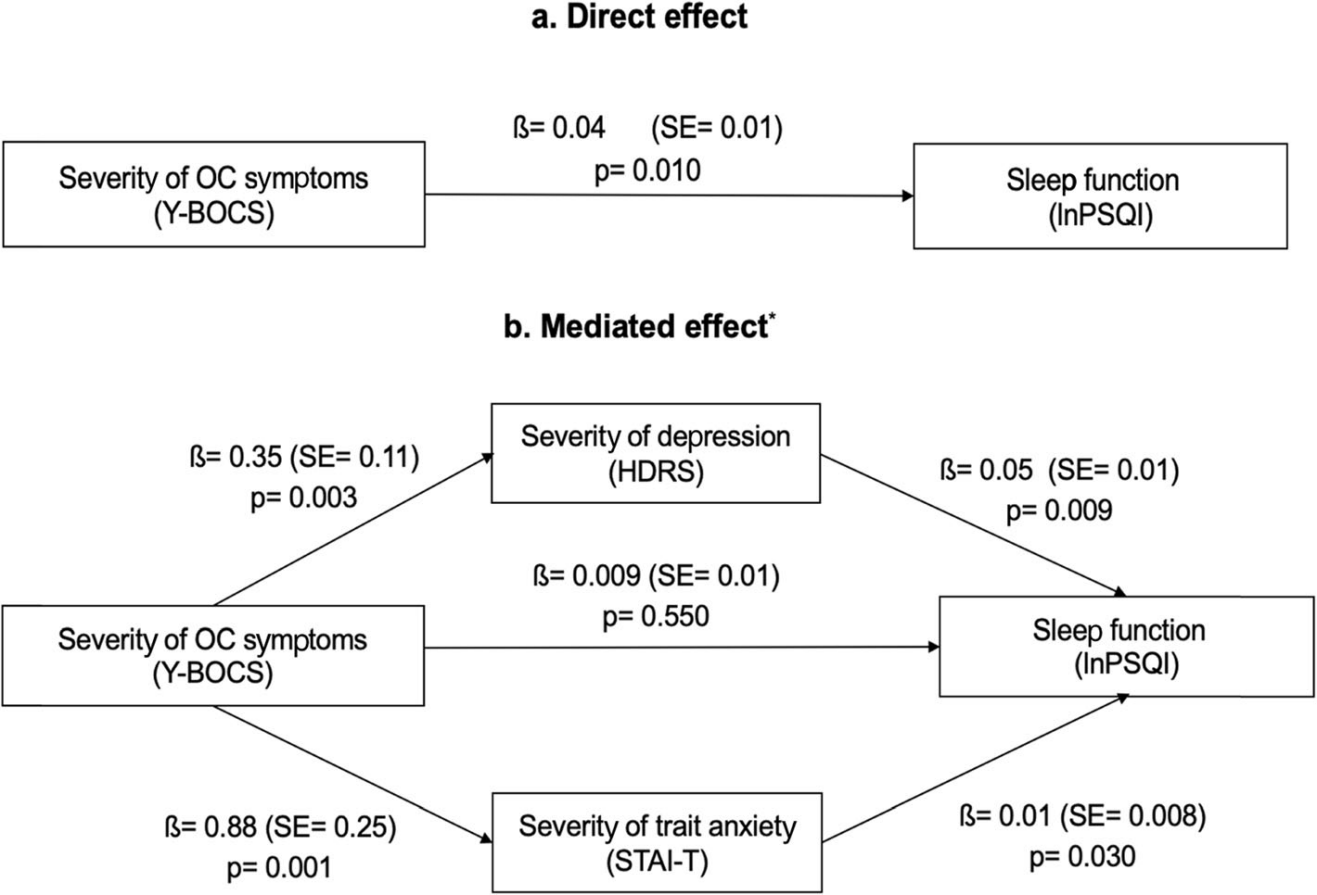
Results of the mediation analysis exploring the relationship between severity of OC symptoms (Y-BOCS) and sleep function (lnPSQI) in subjects with OCD. Log transformed (ln) of sleep function was used in the mediation analysis. (**a**) Unadjusted model. The mediated effect (**b**) was adjusted for age, sex and benzodiazepines doses. Abbreviations: OC= Obsessive Compulsive; Y-BOCS= Yale-Brown Obsessive Compulsive Scale; PSQI= Pittsburgh Sleep Quality Index; HDRS= Hamilton Depression Rating Scale; STAI-T= State-Trait Anxiety Inventory; lnPSQI=Log-transformed (ln) Pittsburgh Sleep Quality Index; β= unstandardized regression coefficient; SE = standard error

In the mediation analyses (Fig. 1), the severity of depression and trait anxiety were analysed as two potential mediators in the relationship between the intensity of OC symptomatology and sleep function. In the unadjusted model (a), the intensity of OC symptoms was positively associated with PSQI scores. This effect was fully mediated by both the severity of trait anxiety and depression (b); similarly, that the relationship between the intensity of OC symptomatology and sleep function was no longer significant when these two mediators were included in the equation. This mediation analysis was adjusted for the following clinical variables: age (β = − 0.003, SE = 0.007, *P* = 0.60), sex (β = − 0.11, SE = 0.15, *P* = 0.47), and benzodiazepine doses (β = 0.005, SE = 0.01, *P* = 0.56). The bootstrapping results for indirect effects were significant for depressive symptoms (95% CI: 0.005 to 0.045) and trait anxiety (95% CI: 0.003 to 0.042).

## Discussion

Patients with OCD reported poorer sleep quality and more sleep disturbances, including a delayed mid-sleep point and a longer time between going to bed and falling asleep, than HCs. Furthermore, the severity of depression and trait anxiety was associated with poorer sleep quality and mediated the relationship between the severity of OC symptoms and poorer sleep quality. Although previous studies had reported the contribution of depressive symptoms to poor sleep quality in OCD, our study is the first to highlight the additional contribution of trait anxiety on sleep quality in OCD patients.

The findings in longer sleep latency, measured as a component of PSQI and registering the time taken to fall asleep, in OCD patients replicate previous studies using self-report measures [9], as well as a meta-analysis of three studies conducted with PSG [10]. On the other hand, the lack of differences in sleep duration and sleep efficiency between both groups appears to contradict other reports with positive results [12–14], although in these studies, variations in sleep duration and sleep efficiency were determined using PSG. Therefore, discrepancies with these findings might be attributable to the use of different instruments to evaluate sleep function, as the PSQI does not correlate well with PSG [28], which is considered the gold standard objective measure of sleep. In addition, a subgroup of OCD patients with comorbid depression reported shorter sleep duration (as measured with the PSQI) than patients with only OCD, thus supporting the influence of depression on sleep duration [9].

The finding that OCD patients have poorer sleep quality than HCs is partially consistent with the results of previous studies that used the same instrument (PSQI) [9, 30]. In contrast, comorbid MDD did not affect poor sleep quality in our study, whereas Bobdey et al. [9] found greater sleep disruption (according to the PSQI) only in OCD patients with comorbid depression compared to patients with only OCD, suggesting that depression substantially influences sleep behaviour.

Multiple systems associated with circadian rhythms are also related to psychopathology, including functions on monoaminergic neurotransmitters, the immune system, and the hypothalamic-pituitary-adrenal axis [31]. Despite the paucity of studies and the inconsistent findings, a link between circadian rhythm sleep disorder (CRSD) and OCD has been described. In this sense, CRSD in OCD is associated with poor treatment response, and some preliminary evidence implicates decreased light exposure and diurnal symptom variability in OCD patients [32]. Our results showed a statistically significant delayed mid-sleep-point and a delay in the getting-up time in the patient group compared to HCs, but there were no differences in the sleep bedtime between both groups. Taking into account that the mid-sleep point is an indirect measure of DSPD, our findings replicate the results of previous studies conducted among patients with OCD [5, 6, 9, 30] and suggest that circadian rhythms may be disrupted in OCD.

In univariate analyses, the severity of trait anxiety and depressive and OC symptoms were positively associated between them and with poor sleep quality. Multivariate analyses suggest that trait anxiety and depressive symptoms (but not OC symptoms) are associated with sleep quality, although the mediation analysis reveals that both psychopathological measures mediate the relationship between OC symptoms and sleep quality. Correlations between trait anxiety and sleep quality have not been previously described in OCD, although associations among depressive symptoms, comorbid MDD and sleep function have been reported by several studies [9–11, 13]. Associations between the severity of OC symptoms and poor sleep quality in our sample are consistent with the findings of previous studies conducted in OCD patients [5, 6]. Our study adds new information regarding the mediating role of trait anxiety and depressive symptoms in this relationship.

Previous studies suggest that several clinical characteristics are associated with CRSD in patients with OCD, including a younger age, early age of onset of OCD, male sex and greater severity of OC symptoms [5, 6]. However, in our sample, we did not find associations between mid-sleep-point (and indirect measure of DSPS) and age, age of onset of OCD, sex or the severity of OC symptoms.

A similar pattern of correlations between clinical variables and sleep quality was found in HCs, with positive associations between trait anxiety and depressive symptoms and poor sleep quality, suggesting a diagnosis-independent relationship. Our findings are consistent with previous studies reporting associations between depressive and anxiety symptoms and sleep disturbances in college student samples [33] . A systematic review of included nine studies conducted in the general population (excluding clinical samples and cohorts at high risk of suffering from sleep disturbances, anxiety and depression) showed a bidirectional relationship between depression/anxiety and several sleep disturbances, suggesting that alterations in sleep behaviour predicts depression/anxiety and vice versa [34]. Strikingly, a delayed mid-sleep point in HCs was negatively correlated with higher global PSQI scores, reflecting poor sleep quality. These findings suggest an association between an indirect measure of CRSD and general sleep quality in HCs. Future studies with larger samples are needed before drawing conclusions regarding this relationship.

Recent findings of neuroimaging studies with functional magnetic resonance imaging (fMRI) reported associations between decreased connectivity of the left amygdala and the bilateral region of the anterior cingulate cortex and increased risk of developing and anxiety disorder in patients with primary insomnia [35]. Regarding depression, increased functional connectivity between different brain areas was associated with both poor sleep quality and depressive problems score [36]. Therefore, novel results in the field of neuroimaging explain the neural mechanism underlying the clinically established associations between sleep function, depression and anxiety. Similarly, findings from a genetic study evaluating whether the polygenic score (PGS) for neuroticism was related to poor sleep quality showed that this effect was mediated by anxiety, depressive symptoms and neuroticism [37]. These results support prior clinical and neuroimaging data and reinforce the biological substrate that might play a role in the relationship between anxiety, depression and sleep behaviour. Results of a National Comorbidity Survey Replication (NCS-R) showed that 90% of patients diagnosed with OCD met the diagnostic criteria for other lifetime disorders in DSM-IV. Amongst these disorders, the most common were anxiety disorders (75.8%), followed by mood disorders (63.3%), impulse-control disorders (55.9%) and substance use disorders (38.6%) [38]. The substantial comorbidity of OCD with anxiety and affective disorders, and the neurobiological relationship established between anxiety, depression and sleep function, suggest that sleep disturbances in OCD could be partly related to the comorbidity with anxiety and depressive disorders. In this regard, a population-based study evaluated the prevalence of insomnia in OCD and determined a significant reduction liability to insomnia when OCD patients with comorbid depression and anxiety disorders were excluded from the analysis [39], suggesting that depressive and anxiety disorders contribute to sleep disturbances in patients with OCD. In line with the previous studies, the fact that the association between poor sleep quality and OC symptoms in the univariate analyses in our sample lost its significance when adjusting for depressive symptoms and trait anxiety suggests that the association with sleep is driven by depressive or anxiety symptoms but not by the severity of OC symptoms.

The limitations of the current study include a limited sample size but are nonetheless larger than previous research using the PSQI to assess sleep function in OCD. We did not include patients with other anxiety or depressive disorders apart from OCD with and without MDD as a clinical comparison group in our study. Its inclusion would have allowed the comparison of sleep patterns between patients suffering from different anxiety and depressive disorders. Our sample included OCD patients recruited from a tertiary care facility, thereby limiting the generalization of these results to community settings. A high percentage of patients (98.4%) was receiving psychopharmacological treatment at the time of assessment, which could have a negative effect on sleep quality, as previous studies have pointed out [4]. In this regard, some antidepressants commonly-prescribed in our sample (Selective Serotonin Reuptake Inhibitors (SSRI) and Tricyclic Antidepressants (TCA)) are sleep-disturbing early [40] and produce alterations in the REM-phase [41]. Although the PSQI is a validated instrument to assess sleep function [28], it is a self-report tool and therefore a nonobjective measure of sleep behaviour. This condition could introduce the possibility of a recall bias. The use of mid-sleep points as an indirect measure of DSPS might be improved by incorporating more extensive and objective measures to assess circadian rhythm, which allows advancement in the study of the relationship between OCD and CRSD. Recommendations to assess CRSD include up to two weeks of actigraphy recording, measurements of endogenous melatonin onset and questionnaires of chronotype [42].

## Conclusions

On the basis of our findings and taking into account the limitations of our study, we can conclude that compared to HCs, patients with OCD show different sleep patterns characterized by poor sleep quality and more sleep disturbances (delayed mid-sleep-point and higher length of time between going to bed and falling asleep). Regarding the influence of clinical variables in sleep function in OCD, our study suggests that the severity of trait anxiety and depression symptoms mediate the negative effect of the intensity of OC symptomatology on sleep quality, indicating that trait anxiety needs to be considered a key element in sleep function, similar to depression. These results need to be confirmed by broader studies with more objective sleep measures to advance the knowledge of sleep behaviour in OCD and to establish better prevention and intervention strategies.

## Supplementary Information

**Additional file 1 Table S1.** Group mean scores on sleep schedule and global for OCD with and without comorbid MDD.

## Abbreviations

OCD: Obsessive-Compulsive Disorder
OC: Obsessive-Compulsive
HCs: Healthy Controls
PSQI: Pittsburg Sleep Quality Index
MDD: Major Depressive Disorder
PSG: Polysomnography
SWS: Slow-Wave Sleep
REM: Rapid Eye Movement
DSPD: Delayed Sleep Phase Disorder
CEIC: Comitè Ètic d’Investigació Clínica
MINI: Mini-International Neuropsychiatric Interview
GHQ: General Health Questionnaire
DDD: Defined Daily Dose
HDRS: Hamilton Depression Rating Scale
STAI: State-Trait Anxiety Inventory
Y-BOCS: Yale-Brown Obsessive Compulsive Scale
SPS: Sleep Phase Shifting
CI: Confidence Interval
ln: Log transformed
CRSD: Circadian Rhythm Sleep Disorder
fMRI: Functional Magnetic Resonance Imaging
PGS: Polygenic Score
NCS-R: National Comorbidity Survey Replication (RNS-R)
SSRI: Selective Serotonin Reuptake Inhibitors
TCA: Tricyclic Antidepressants
