# Supplementary material for: Sleep disturbances in obsessive-compulsive disorder: influence of depression symptoms and trait anxiety
Source: BMC Psychiatry. 2021 Jan 14;21:42. doi: 10.1186/s12888-021-03038-z (PMC7809865; doi:10.1186/s12888-021-03038-z)
Supplement: Supplementary file 1 — Additional file 1 Table S1. Group mean scores on sleep schedule and global for OCD with and without comorbid MDD. [file 12888_2021_3038_MOESM1_ESM.docx]

**Supplementary Table 1.** Group mean scores on sleep schedule and global for OCD with and without comorbid MDD

|  | OCD + MDD | OCD |  |  |  |
| --- | --- | --- | --- | --- | --- |
|  | N % | N % |  |  |  |
|  | 26 42.6 | 35 57.4 |  |  |  |
|  | Mean (*SD)* | Mean (*SD)* | Statistic | df | *P* value |
| PSQI GS | 9.62 (5.2) | 7.63 (4.44) | *t*=-1.6 | 59 | 0.12 |
| Time falling sleep (min) | 45.85 (45:21) | 28:14 (28:32) | *t*=-1.87 | 59 | 0.06 |
| Sleep bedtime (h:min) | 24:22 (1:19) | 24:12 (1:31) | *t*=-0.47 | 59 | 0.64 |
| Getting-up bedtime (h:min) | 8:55 (1:48) | 8:30 (1:40) | *t*=-0.95 | 59 | 0.34 |
| Mid-sleep-point (h:min) | 4:42 (1:21) | 4:22 (1:24) | *t*=-0.95 | 59 | 0.34 |

Abbreviations: OCD, Obsessive-Compulsive Disorder; MDD, Major Depressive Disorder; HC, Healthy Controls; *SD*, Standard Deviation; PSQI GS, Pittsburgh Sleep Quality Index Global Score; h, hour; min, minutes.**P<*0.05.
